# Impact of Perineuronal Net Removal in the Rat Medial Prefrontal Cortex on Parvalbumin Interneurons After Reinstatement of Cocaine Conditioned Place Preference

**DOI:** 10.3389/fncel.2022.932391

**Published:** 2022-07-28

**Authors:** Angela E. Gonzalez, Emily T. Jorgensen, Jonathan D. Ramos, John H. Harkness, Jake A. Aadland, Travis E. Brown, Barbara A. Sorg

**Affiliations:** ^1^Program in Integrative Physiology and Neuroscience, Washington State University, Vancouver, WA, United States; ^2^Dow Neurobiology Laboratories, Legacy Research Institute, Portland, OR, United States; ^3^Neuroscience Graduate Program and School of Pharmacy, University of Wyoming, Laramie, WY, United States; ^4^Rewire Neuro, Portland, OR, United States

**Keywords:** addiction, cocaine, conditioned place preference, memory, parvalbumin, perineuronal nets, prefrontal cortex, substance use disorder

## Abstract

Parvalbumin (PV)-positive cells are GABAergic fast-spiking interneurons that modulate the activity of pyramidal neurons in the medial prefrontal cortex (mPFC) and their output to brain areas associated with learning and memory. The majority of PV cells within the mPFC are surrounded by a specialized extracellular matrix structure called the perineuronal net (PNN). We have shown that removal of PNNs with the enzyme chondroitinase-ABC (Ch-ABC) in the mPFC prevents the consolidation and reconsolidation of cocaine-associated conditioned place preference (CPP) memories. Here we examined the extent to which retrieval of a CPP memory during cocaine-primed reinstatement altered the levels and function of PV neurons and their surrounding PNNs during the reconsolidation period. We further determined the extent to which PNN removal prior to reinstatement altered PV intensity levels and PV cell function. Male Sprague-Dawley rats were trained for cocaine-induced conditioned place preference (CPP) followed by extinction training, microinjection of Ch-ABC in the prelimbic PFC, and cocaine-induced reinstatement. Rats were sacrificed immediately prior to reinstatement or at 2 h, 6 h, or 48 h after reinstatement for immunohistochemistry or 2 h later for electrophysiology. Our findings indicate that PNN removal only partially diminished reinstatement. Cocaine-primed reinstatement produced only minor changes in PNN or PV intensity in vehicle controls. However, after PNN removal, the intensity of remaining PNN-surrounded PV cells was decreased at all times except at 2 h post-reinstatement, at which time cocaine increased PV intensity. Consistent with this, in vehicle controls, PV neurons naturally devoid of PNNs showed a similar pattern to Ch-ABC-treated rats prior to and after cocaine reinstatement, suggesting a protective effect of PNNs on cocaine-induced changes in PV intensity. Using whole-cell patch-clamp, cocaine-primed reinstatement in Ch-ABC-treated rats decreased the number of elicited action potentials but increased excitatory synaptic transmission, which may have been compensatory. These findings suggest that without PNNs, cocaine-induced reinstatement produces rapid changes in PV intensity and PV cell excitability, which may in turn regulate output of the mPFC post-memory retrieval and diminish the maintenance of cocaine memory during reconsolidation.

## Introduction

One of the major problems confronting the treatment of cocaine use disorder is the high propensity for relapse. Cues and contexts associated with drug use, such as drug paraphernalia or the drug-taking environment, can elicit the recall of past experiences that drive craving and relapse (Volkow et al., 2002). The medial prefrontal cortex (mPFC) is heavily implicated in the reinstatement of cocaine seeking in rodents (Mcfarland and Kalivas, 2001; Park et al., 2002; Capriles et al., 2003; Mclaughlin and See, 2003; Mcfarland et al., 2004; Fuchs et al., 2007; Peters et al., 2008) and cocaine relapse in humans (Volkow et al., 1991, 1996; Grant et al., 1996; Maas et al., 1998; Childress et al., 1999). Pharmacological and optogenetic studies show that inactivation of the prelimbic mPFC blocks cue-induced (Mclaughlin and See, 2003), cocaine-induced, cocaine + cue-induced (Mcfarland and Kalivas, 2001; Stefanik et al., 2013), and stress-induced (Capriles et al., 2003) reinstatement in self-administering rats.

The output of pyramidal neurons in the mPFC is powerfully modulated by parvalbumin-containing (PV) interneurons (Markram et al., 2004; Sparta et al., 2014), which themselves receive dopamine input and are thus ideally situated to regulate mPFC projections to the nucleus accumbens (Benes et al., 1993; Seamans et al., 2001) and may contribute to cocaine-induced plasticity (Jayaram and Steketee, 2005; Kroener and Lavin, 2010; Campanac and Hoffman, 2013). The majority of PV neurons in the cortex are encased in a specialized extracellular matrix called the perineuronal net (PNN). PNNs form in the central nervous system during the critical period of development and stabilize neural networks in adulthood (Fawcett et al., 2019). PNNs allow PV neurons to generate fast-spiking activity needed for precise firing during gamma oscillations, which underlie attention (Kim et al., 2016; Bueno-Junior et al., 2017), adapting new strategies (Guise and Shapiro, 2017; Cho et al., 2020), and encoding reward outcomes (Donnelly et al., 2014).

Reconsolidation is a memory maintenance process in which memories are updated and stabilized, including strong drug-associated memories that are difficult to disrupt (Lee et al., 2005; Sorg, 2012; Tronson and Taylor, 2013). Reconsolidation allows for integration of new information within a specific time window following memory retrieval. During this time, a molecular cascade of events initiates processes that last about 6 h before the window closes and the memory is no longer labile (Nader et al., 2000). PNNs are thought to contribute to the maintenance of memories through their network stabilizing properties (Fawcett et al., 2019) and may do so through dynamic changes in PNNs and their underlying PV neurons during the reconsolidation window.

We previously demonstrated that enzymatically degrading PNNs in the dorsal mPFC (mainly prelimbic PFC) with chondroitinase ABC (Ch-ABC) reduced the acquisition and reconsolidation of cocaine-induced conditioned place preference (CPP) in rats (Slaker et al., 2015). In a follow-up study, we tested whether PNNs and their underlying PV neurons were altered after a cocaine memory retrieval session in which rats were placed back into the CPP environment in a drug-free state (Jorgensen et al., 2020). The intensity of PV rapidly decreased beginning 30 min after the CPP test, and this decrease was retained for up to 24 h, with no change in the intensity or number of PNNs in the mPFC (Jorgensen et al., 2020). Additionally, the intrinsic excitability of PNN-surrounded PV cells was decreased and accompanied by increases in excitatory input, as determined by an increased frequency of mEPSCs and VGluT1 puncta apposing PV/PNN cells. These findings suggested that PV neuron function was altered by a drug-free memory retrieval session. In addition, we reported that when given alone, cocaine CPP training or enzymatic PNN removal reduced the frequency of mIPSCs onto pyramidal neurons in the prelimbic PFC and that PNN removal increased the excitability of pyramidal neurons (Slaker et al., 2015). However, we did not test the impact of cocaine-primed reinstatement on PV neuron function, which is important because the presence of cocaine at the time of retrieval is likely to provide interoceptive cues that enhance memory retrieval. It is also unknown if PNNs and PV neurons are involved in cocaine-primed reinstatement. Based on our previous finding that PNNs serve a key role in the reconsolidation of cocaine-induced CPP (Slaker et al., 2015), in the current study, we obtained a detailed profile of PV neuron changes after cocaine-primed memory retrieval during the period of time over which memory reconsolidation processes occur and 48 h later in the absence and presence of PNNs.

## Materials and Methods

### Animals

A total of 71 male Sprague-Dawley rats was used in this study. Of these 71 rats, 47 rats weighing 275–300 g at the start of the experiment were obtained from Simonsen Laboratories (Gilroy, CA) or Envigo (Livermore, CA) (behavior and immunohistochemistry), and a total of 24 male Sprague-Dawley rats were bred in-house at the University of Wyoming (behavior and electrophysiology). Animals were singly housed in a temperature and humidity-controlled room maintained on a 12-hour light/dark cycle, with lights-on at 0700, and all experiments were conducted during the light cycle. Except for daily CPP and behavioral testing, animals had access to food and water *ad libitum* throughout the study. Rats were habituated to housing conditions and handled for 1 week prior to surgery. All experiments were approved by the Institutional Animal Care and Use Committees at Washington State University, Legacy Research Institute, and The University of Wyoming in accordance with the National Institute of Health's *Guide for the Care and Use of Laboratory Animals*. All efforts were made to minimize the number of animals used in the experiments and to reduce the amount of pain and suffering.

### Surgery

Animals weighing ~300 g were anesthetized with ketamine/xylazine (87 mg/mL/13 mg/mL) dissolved in saline; intramuscular) or ketamine/dexmetetomidine [75 mg/kg/1 mg/kg in saline; intraperitoneal (i.p.)] and placed in a stereotaxic apparatus. The prelimbic mPFC was targeted with 12 mm surgical-grade steel cannula (26 gauge) (+3.2 mm A/P, +0.8 mm M/L, and−2.5 D/V) (Paxinos and Watson, 2007) and fixed with dental acrylic cement. Obturators (33 gauge) measuring the length of the guide cannulae were inserted into the cannulae following surgery and remained in place until the time of microinjection. Rats were given 3 days of post-operative analgesics (2 mg Rimadyl tablets and subcutaneous buprenorphine (0.05 mg/kg, subcutaneous, twice daily), carprofen (5 mg/kg/day, MediGel), or ketaprofen (10 mg/kg intramuscularly along with antibiotic treatment (Baytril tablets (2 mg/tablet) in Sucralose Medi-gel cups.

### Drugs

Cocaine-HCl (gift from the National Institutes of Health) was dissolved in sterile saline. For CPP training, a dose of 12 mg/kg was injected intraperitoneally (i.p.), and for the cocaine-induced reinstatement session, a dose of 10 mg/kg, i.p. was injected. Protease-free chondroitinase-ABC (Ch-ABC) was obtained from Sigma-Aldrich and dissolved in sterile saline to a final concentration of 0.09 U/μL, and 0.4–0.6 μL/hemisphere was delivered bilaterally.

### Cocaine-Conditioned Place Preference

All behavioral training and testing were performed during the animals' light phase. CPP chambers from Med Associates consisted of three distinguishable compartments separated by manual guillotine doors as previously described (Jorgensen et al., 2020). Locomotion and chamber preference were automatically detected by infrared photocell beams throughout the three chambers and recorded using Med Associates software.

Animals were habituated to the CPP apparatus for 2 days prior to training. The second exposure day was used to determine initial preference to either of the primary chambers and was measured by time spent in each chamber. During the habituation days, animals were placed in the central chamber with open access to all chambers for 15 min. After the second habituation day (initial preference day), rats were counterbalanced within each cohort for pairing of cocaine with the black or white chamber and for the preferred or non-preferred chamber. Animals spending 400 s or more in one chamber during the initial preference test were automatically assigned to the non-preferred chamber for cocaine injection during the training phase.

Cocaine and saline training consisted of six consecutive days of injections. Cocaine (12 mg/kg, i.p.) and saline (1 mL/kg, i.p.) were administered on alternating days and paired with the assigned chamber, each rat receiving three cocaine and three saline pairings. On each day of training, animals were weighed, injected, and confined to the assigned chamber for 25 min. Cocaine place preference was then determined in a drug-free state on the day following the last training day (test day). On test day, animals were placed in the center chamber with access to all chambers for 15 min. Side preference was determined when the amount of time spent in the cocaine-paired chamber in a cocaine-free state was higher than the initial preference day. Animals that did not attain place preference were not included in the study. Following testing, animals were then given extinction sessions to extinguish the cocaine-preferred context for 8–12 days. Extinction sessions consisted of 25-min, cocaine-free sessions with access to all three compartments. Rats were considered extinguished when time spent on the cocaine paired side for each cohort was not significantly different from their initial preference day.

On the last extinction day, 4 h post extinction, animals were microinjected with vehicle or Ch-ABC. After 72 h post injection, one group of rats was euthanized to obtain pre-reinstatement measurements prior to cocaine-induced reinstatement (pre-reinstatement group), while three additional groups were given a cocaine-induced reinstatement session and euthanized at various time points (t = 2 h for electrophysiology, and t = 2 h, 6 h, or 48 h for IHC analyses). These time points for IHC were chosen based on our previous study showing an impact of cocaine CPP memory retrieval in the drug-free state at 2 h and, in some cases, at 24 h after memory retrieval (Jorgensen et al., 2020). Thus, we added a 48 h time point to see if effects persisted past 24 h. We additionally chose the 6 h timepoint in the current study based on observations that the reconsolidation window is ~6 h (Nader et al., 2000). For electrophysiology studies, we focused on the 2 h timepoint based on our previously observed changes in PV neuron firing after CPP memory retrieval (Jorgensen et al., 2020). Reinstatement consisted of a cocaine injection (10 mg/kg, i.p.) followed by immediate placement into the center chamber and with access to all three chambers for 15 min. Time spent on the cocaine-paired side on reinstatement day was compared to initial preference day and the last extinction day. For euthanasia, animals were perfused intracardially with 4% paraformaldehyde (PFA) in 0.1 M phosphate buffered saline (PBS). The brains were removed and stored overnight in 4% PFA at 4°C. The next day, brains were moved to a 20% sucrose in 0.1 M PBS solution; and 48 h later, brains were flash-frozen and stored at−80°C until collection of mPFC tissue sections.

### Intracranial Microinjection

For microinjections, a surgical-grade steel needle (13 mm; 33 gauge) was connected to tubing attached to a 1.0 Hamilton syringe and inserted into the cannula. A volume of 0.4 to 0.6 μL was injected into each hemisphere over a period of 2 min. Following injection, the needles remained in place for an additional 1 min to allow for the enzyme to diffuse. Microinjection spread and cannulae placement were verified with histology and confocal imaging. All animals with bilateral cannulae placement and enzyme degradation contained within the majority of the prelimbic PFC were included in analysis.

### Immunohistochemistry and Microscopy

Using a freezing microtome, 40 μm coronal brain sections of the prelimbic PFC were collected from +4.4 mm to +3.0 mm A/P and stored at 4°C in a storage buffer solution consisting of 25% glycerol, 25% ethylene glycol and 50% PBS. For IHC, tissue sections were rinsed three times for 5 min in PBS. Tissue was then placed in a blocking solution made of 5% BSA (Sigma), 1-X PBS, and 0.25% Triton-X for 1 h. After rinsing an additional three times for 5 min in PBS, tissue was incubated in 1% sodium borohydride for 30 min and rinsed three times for 5 min in PBS before an overnight incubation at 4°C in mouse anti-parvalbumin monoclonal antibody (1:1000, Sigma-Aldrich Cat# SAB4200545, RRID:AB_2857970), 2% BSA, 0.25% Triton-X in PBS. The following day, tissue was rinsed three times for 5 min in 1-X PBS and placed in 1:500 dilution of AlexaFluor goat anti-mouse 594 (Abcam Cat# ab150116, RRID:AB_2650601), 2% BSA, 0.25% Triton-X in PBS for overnight incubation at 4°C. On the last day, tissue was rinsed three times for 5 min and incubated in *Wisteria floribunda* agglutinin (WFA, 1:500, Vector Laboratories Cat# FL-1351, RRID:AB_2336875), 2% BSA, 0.25% Triton-X in PBS for 2 h and rinsed an additional three times. Brain sections were then mounted onto FrostPlus slides in diluted PBS with 0.15% Triton-X and coverslipped prior to tissue dehydration with ProLong Gold Antifade reagent (Vector Laboratories).

Images of the prelimbic PFC were collected using a Leica SP8 laser scanning confocal microscope with an HCX PL apo CS, dry, 40x objective with 0.70 numerical aperture. Calibration of the laser intensity, gain, offset, and pinhole settings were determined using a section of tissue containing intact PNNs in the prelimbic PFC. The same settings were maintained for all images. Four images per animal were collected in z-stacks of 20 images each (step size 0.44 μm; containing the middle 8.8 μm of each 40 μm section). Analysis of PV and WFA stained cells was confined to layers 5/6, since we wished to target PV basket cells (vs. chandelier cells), as only basket cells have PNNs, as measured with *Vicia villosa* agglutinin (Naegele and Katz, 1990; Ariza et al., 2018). Basket cells but not chandelier cells are found in layers 5/6 of the mPFC (Miyamae et al., 2017).

All images (1.194 pixels/μm) were compiled into summed images using ImageJ macro plug-in Pipsqueak™ (https://rewireneuro.com/pipsqueak-pro/) (RRID:SCR_022148), scaled, and converted into 8-bit, grayscale, tiff files. Pipsqueak™ was used to identify and quantify the intensity signal and double-labeled parvalbumin and WFA-labeled cells with the “double-label analysis” function in “semi-automatic mode”. The resulting txt. files from Pipsqueak™ were then processed for intensity and cell count. This was performed by preprocessing and normalizing data to control animals (pre-reinstatement time point for vehicle control rats). Tissue sections were examined for rostral to caudal cannula placement and spread of Ch-ABC after imaging for intensity analysis to prevent bleaching. Animals without appropriate placement or degradation of PNNs within the prelimbic PFC (visualized with the WFA stain) were not included in imaging or behavioral analysis.

### Whole-Cell Patch Clamp Electrophysiology

For whole-cell patch clamp, tissue preparation and solution compositions were as previously described (Slaker et al., 2018; Jorgensen et al., 2020). Following perfusion, rats were decapitated and coronal slices (300 μm) containing the mPFC were prepared using a vibratome (Leica VT1200S), placed in ice-cold recovery solution, and incubated for at least 1 h in room temperature, in a modified aCSF holding solution prior to recording. Immediately before recording, slices were incubated in holding solution WFA (1 μg/mL) for 5 min to stain for PNNs. CellSens software (Olympus) was used to identify WFA (fluorescing) neurons in layers 5/6 of the prelimbic mPFC and later patched. Pipettes were filled with K-Gluconate intracellular solution for intrinsic experiments. K-Gluconate composition was (in mM): 120 K-Gluconate, 6 KCl, 10 HEPES, 4 ATP-Mg, 0.3 GTP-Na, 0.1 EGTA, and KOH was added to bring pH to ~7.2. Intrinsic properties of cells were recorded in current-clamp (no holding current applied). 10 current steps were injected starting at −100 pA and ending at 500 pA. The duration of each recording was no longer than 5 min per cell with each sweep lasting 100 ms. Elicited action potentials were recorded, counted, and analyzed using pClamp10.3. (Clampfit, Axon Instruments, Sunnyvale, CA). Multiple cells from a single animal were collapsed into a single data point for representation (**Figure 4**) and analysis.

Miniature excitatory postsynaptic currents (mEPSCs) were recorded in an aCSF containing picrotoxin (100 μM) and tetrodotoxin (1 μM) to block GABA-A receptors and sodium channels, respectively. Patch pipettes were filled with KCl intracellular solution. KCl solution composition was (in mM): 125 KCL, 2.8 NaCl, 2 MgCl_2_, 2 ATP-Na^+^, 0.3 GTP-Li^+^, 0.6 EGTA, and 10 HEPES. Cells were voltage-clamped at −70 mV and input resistance and series resistance were monitored throughout experiments. Miniature recordings were 3 min long with 1 min per sweep. Only one sweep per cell was used for data analysis. Criteria were met if the cell's access resistance was below 30 MΩ. mEPSCs were amplified and recorded using pClamp10.3, and Mini Analysis Program (Synaptosoft Inc, GA USA) was used to measure miniature amplitudes and frequencies.

### Statistics

The statistics for behavior, immunohistochemistry, and electrophysiology were performed using GraphPad Prism 9 software. Behavioral experiments were analyzed using a two-way ANOVA followed by Šídák's multiple comparisons test for within-subjects comparison across days and between-subjects comparisons for treatment (vehicle vs. Ch-ABC). Analysis of PV and WFA intensity were log-transformed due to their non-normal distribution and analyzed using a two-way ANOVA. Cell number analysis was performed using a two-way ANOVA followed by Šídák's multiple comparisons test and are shown as bar graphs with individual cells (circles). For electrophysiological analysis to analyze frequency of action potentials, two-way ANOVA followed by Šídák's multiple comparisons test was performed and are shown as bar graphs with individual cells (circles). For intrinsic properties, data were analyzed with two-tailed *t* tests and corrected with Welch's *t* test where appropriate. All results are summarized as the mean of the data set (bar graphs) with a scatter plot of the individual cells (circles). For miniature electrophysiological analysis, the first 300 events were analyzed for amplitude analysis and the first 50 events were analyzed for frequency analysis, data were then analyzed with a Kolmogorov-Smirnov test for cumulative probability distributions. Differences were considered significant if p < 0.05. The data that support the findings of this study are available upon request from the corresponding author.

## Results

### PNN Removal Has Minimal Impact on Cocaine-Primed Reinstatement

**Figure 1:**
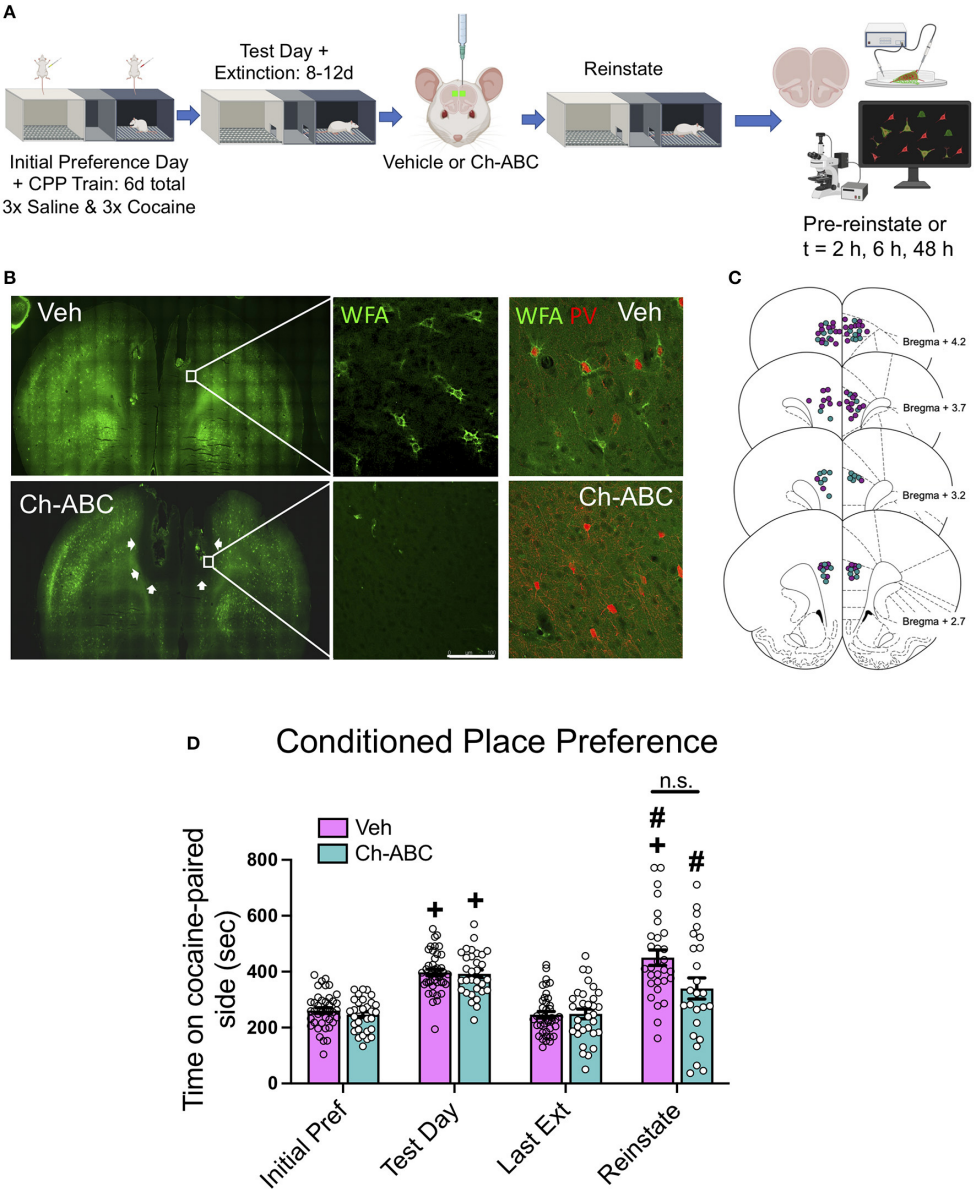
PNN removal in the mPFC has minimal impact on cocaine-primed reinstatement. **(A)** Experimental timeline for cocaine-induced CPP training, testing, and reinstatement. **(B)** (Top panels) Representative images of WFA-stained coronal sections of mPFC of vehicle and Ch-ABC-treated rat. White line = 100 μm. Upper right panel: WFA/PV double-labeled cells in vehicle-treated rat; Lower right panel: WFA/PV double-labeled cells in Ch-ABC-treated rat; note lack of WFA surrounding PV cells. **(C)** Schematic diagram (Paxinos and Watson, 2007) of mPFC coronal rat brain sections depicting stereotaxic coordinates of vehicle and Ch-ABC microinjections; purple, vehicle; teal, Ch-ABC. **(D)** There were no differences in treatment within subjects on initial preference (Initial Pref) or test day. Both treatment groups showed cocaine CPP on test day compared to their initial preference day within subjects. Both treatment groups also showed cocaine-induced reinstatement compared to their last extinction day (Last Ext). Vehicle subjects showed increased preference on reinstatement day (Reinstate) compared to their initial preference day. Data are mean ± SEM. For vehicle, n = 40; Ch-ABC n = 31 rats. ^+^*P* < 0.05 compared with initial preference day; ^#^*P* < 0.05, compared with last extinction day. Created with BioRender.com.

Figure 1A shows the experimental timeline for training, testing, microinjection, and cocaine-primed reinstatement. Figure 1B shows that microinjection of the enzyme Ch-ABC into the prelimbic PFC degraded PNNs. Figure 1C shows the location of injection sites for all animals. Note that Ch-ABC effects encompass areas dorsal to the prelimbic PFC due to its spread along the implanted chronic guide cannulae. Figure 1D shows behavior over all days for all rats during the training and testing for cocaine-induced CPP. A two-way ANOVA revealed that there was no main effect of treatment [F (1,45) = 3.0305, p = 0.0757], but a main effect of day [F(1.613, 72.6) = 37.42, <0.0001], and no treatment x day interaction [F(3,135) = 1.789, p = 0.1522]. Šídák's multiple comparisons test showed an increase in CPP on the test day compared to initial preference day (vehicle, *p* < 0.0001; Ch-ABC, *p* < 0.0001), and a decrease in CPP on the last extinction day compared to the test day (Vehicle, *p* < 0.0001; Ch-ABC, *p* < 0.0001). Cocaine-induced reinstatement was observed in both vehicle (*p* < 0.0001) and Ch-ABC (*p* = 0.0009) -treated animals compared to their last extinction day. Within subjects, CPP on the reinstatement day increased in vehicle-treated animals compared with their initial preference day (*p* < 0.0001), with no significant reinstatement in Ch-ABC-treated animals compared to their initial preference day (*p* = 0.0653). Reinstatement was not different between vehicle- and Ch-ABC-treated rats (*p* = 0.0892). There was no difference between vehicle and Ch-ABC treatment on locomotor activity on any of the training or testing days (not shown). These findings suggest that PNN removal 3 days prior to reinstatement but after CPP training and extinction did not have a major impact on cocaine-induced CPP reinstatement.

### PNN Removal Increases PV Intensity 2 h After Cocaine-Primed Reinstatement

**Figure 2:**
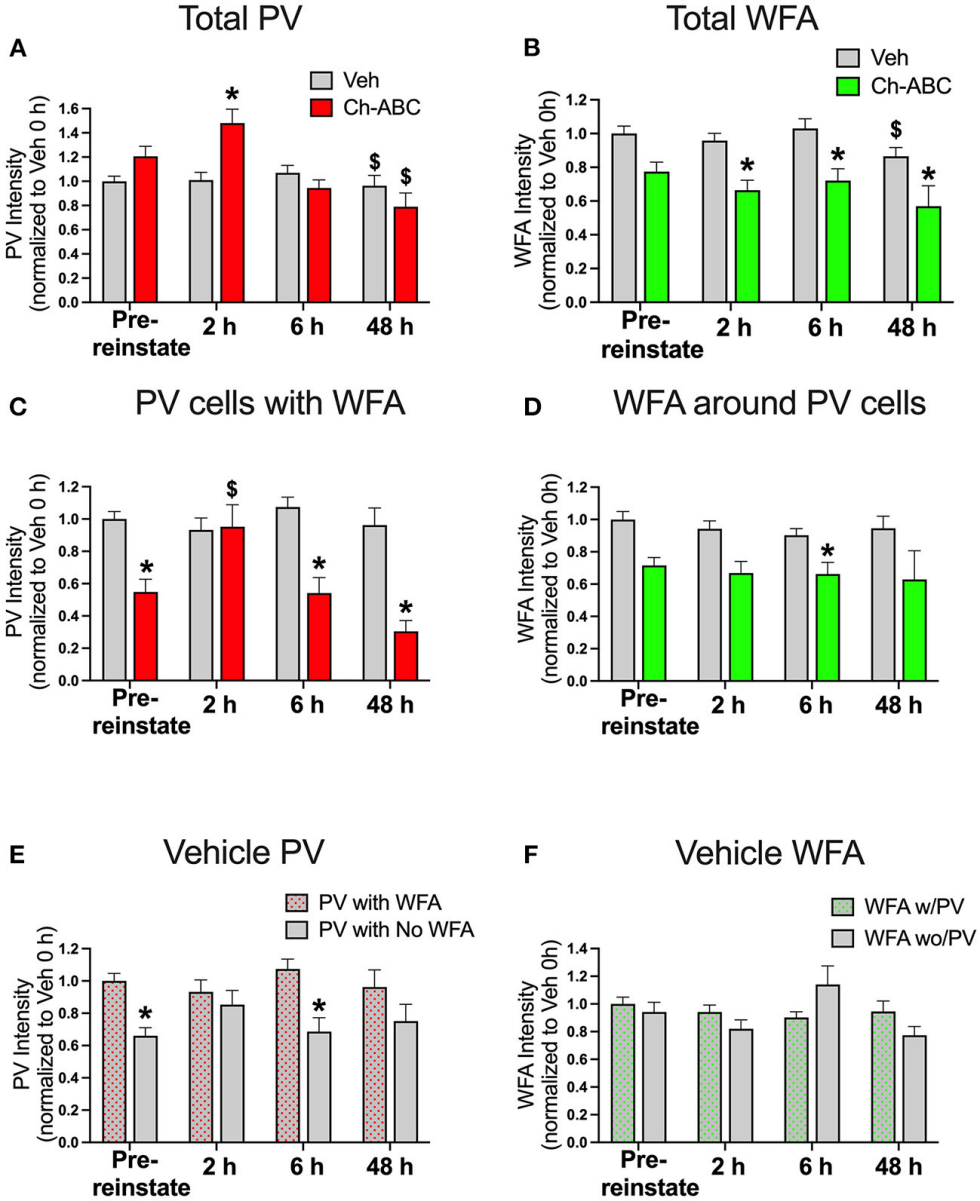
Cocaine-primed reinstatement does not alter PV or WFA intensity in vehicle controls, while Ch-ABC treatment in the mPFC increases PV intensity 2 h post-reinstatement. **(A)** Intensity of total labeled PV cells in prelimbic PFC; PV intensity of both vehicle and Ch-ABC-treated rats decreased at 48 h compared within group to intensity at pre-reinstatement (pre-reinstate); intensity of Ch-ABC-treated animals increased at 2 h compared to vehicle-treated animals. **(B)** Intensity of total labeled WFA cells in prelimbic PFC; WFA intensity in Ch-ABC-treated animals decreased at 2, 6, and 48 h compared to vehicle treated animals; intensity of WFA in vehicle-treated animals at 48 h decreased compared to their pre-reinstatement levels. **(C)** Intensity of PV cells containing WFA in prelimbic PFC; intensity of PV cells in Ch-ABC-treated animals was decreased at pre-reinstatement and at 6 and 48 h compared to vehicle-treated animals; intensity of PV cells in Ch-ABC-treated animals increased at 2 h compared to pre-reinstatement levels in Ch-ABC-treated animals. **(D)** Intensity of WFA cells in prelimbic PFC containing PV; intensity of WFA in Ch-ABC-treated animals decreased at 6 h compared to vehicle-treated animals. **(E)** Intensity of PV with or without WFA in prelimbic PFC in vehicle groups; intensity of PV devoid of PNNs (without WFA) decreased at pre-reinstatement and at 6 h compared to PV cells with PNNs (with WFA). **(F)** Intensity of WFA with or without co-labeled PV in prelimbic PFC. Data are mean ± SEM. All values were normalized to vehicle pre-reinstatement values. For vehicle: pre-reinstate n = 10; 2 h n = 7; 6 h n = 6; 48 h n = 5. For Ch-ABC: pre-reinstate n = 6; 2 h n = 6; 6 h n = 5; 48 h n = 2. ^$^*P* < 0.05, compared within-group to Pre-reinstate; *P < 0.05 compared to vehicle group at the same time point.

Our previous work showed that CPP memory retrieval alone in the drug-free state decreased PV intensity for up to 24 h (Jorgensen et al., 2020). Therefore, we examined the impact of cocaine-primed reinstatement on PV and PNN intensity prior to reinstatement (pre-reinstatement) and at intervals after reinstatement (2, 6, and 48 h post cocaine). All groups were normalized to pre-reinstatement levels of staining for PNNs and PV in vehicle-treated rats. Figure 2A shows the impact of reinstatement on the intensity of all PV cells (total PV intensity). A two-way ANOVA showed no effect of treatment [F(1,1242) = 0.2097, P = 0.6471], but an effect of time [F(3,1242) = 7.903, *p* < 0.0001] and a treatment x time interaction [F(3,1242) = 4.135, p = 0.0063]. Within treatment groups, there was a decrease in PV intensity at 48 h post-reinstatement in rats given both vehicle (*p* = 0.0018) and Ch-ABC (*p* = 0.0079) compared with their own pre-reinstatement intensities. Between groups, rats given Ch-ABC vs. vehicle increased PV intensity 2 h after reinstatement (*p* = 0.0132). Figure 2B shows the intensity of all WFA-labeled cells (total WFA intensity). A two-way ANOVA showed a main effect of treatment [F(1,749) = 29.48, *p* < 0.0001] and a main effect of time [F(3,749) = 2.879, p = 0.0352], but no treatment x time interaction [F(3,749) = 0.9001, *p* = 0.4407]. Within treatment groups, there was a decrease in WFA intensity at 48 h post-reinstatement in rats given vehicle (*p* = 0.0327) compared with pre-reinstatement, but no changes across time in rats given Ch-ABC. Between groups, rats given Ch-ABC vs. vehicle decreased PNN intensity at all three times post-reinstatement (2 h, *p* = 0.0137; 6 h; *p* = 0.0004; 48 h p = 0.0465), as expected after degradation of PNNs.

We also examined the intensity of double-labeled PV/WFA cells, which are shown in Figure 2C. For PNN-surrounded PV cells, a two-way ANOVA showed a main effect of treatment [F(1,521) = 21.58, *p* < 0.0001], time [F(3,521) = 3.511, p = 0.0152], and treatment x time interaction (F(3,521) = 4.470, p = 0.0041). Within treatment groups, there were no changes in PV intensity across time in vehicle-treated rats, but there was an increase in intensity of PV/WFA co-labeled cells in Ch-ABC-treated rats 2 h post-reinstatement (*p* = 0.0202) compared with their pre-reinstatement intensity. Between groups, we observed a decrease in PV intensity in Ch-ABC vs. vehicle rats at pre-reinstatement (*p* < 0.0001), at 6 h (*p* < 0.0001), and 48 h (*p* = 0.0321). We also measured the intensity of WFA surrounding PV cells, shown in Figure 2D. A two-way ANOVA revealed a main effect of treatment [F(1,521) = 19.05, *p* < 0.0001], but no effect of time [F(1,521) = 1.106, *p* = 0.3463] or treatment x time interaction [F(3,521) = 0.412, *p* = 0.7441]. We found that rats given Ch-ABC decreased PNN intensity around PV cells 6 h post-reinstatement compared to rats given vehicle (*p* = 0.0009), with no change at pre-reinstatement (*p* = 0.0918).

### Cocaine-Primed Reinstatement Alters Intensity of PV Cells Devoid of PNNs in Vehicle Controls

Although we did not find changes in the intensity of total PV or of PV cells surrounded by PNNs in vehicle-treated animals (Figure 2C), we wished to also examine whether the presence of cocaine during memory reactivation would impact PV intensity in PV cells naturally with vs. without PNNs (Figure 2E) or WFA intensity in PNNs naturally surrounding PV vs. non-PV cells (Figure 2F) in vehicle control rats. Using a 2-way ANOVA, we found a main effect of treatment [F(1,763) = 32.61, *p* < 0.0001] but no effect of time [F(3,736) = 2.116, *p* = 0.0968], and no effect of treatment x time interaction [F(3,763) = 2.016, *p* = 0.1103]. Between groups, the intensity of PV cells without WFA was decreased compared to PV cells with WFA at pre-reinstatement (*p* < 0.0001) and at 6 h post-reinstatement (*p* = 0.0001) (Figure 2E). Combined with the absence of changes in PV cells surrounded by PNNs after cocaine-reinstatement (Figure 2C), these findings show that PV intensity is generally greater in PNN-surrounded cells and suggest that PNNs may protect the reduction in PV levels found after cocaine CPP training (pre-reinstatement levels), and also at 6 h post-reinstatement.

Figure 2F shows that the intensity of WFA around PV cells vs. non-PV cells was not altered, as there was no main effect of treatment [F(1,684) = 2.456, *p* = 0.1175], a strong trend toward an effect of time [F(3,684) = 2.588, p = 0.052], and no effect of treatment x time interaction [F(3,684) = 1.477, *p* = 0.2196]. In vehicle groups, there was no impact of cocaine-induced reinstatement on the intensity of WFA surrounding PV cells compared to the intensity of WFA surrounding non-PV cells (Figure 2F).

Overall, our findings examining intensity of PV showed a lack of change in vehicle-treated rats with exception of a slight decrease at 48 h post-cocaine-primed reinstatement, which generally did not mimic the early decreases we previously found after CPP testing in the drug-free state (Jorgensen et al., 2020). In Ch-ABC-treated rats, the decrease in PV intensity after Ch-ABC is expected based on previous work (Yamada et al., 2014), but the increase in PV intensity at 2 h post-reinstatement was unexpected and suggests that either the pharmacological effects of cocaine and/or the cocaine memory in the added presence of internal cocaine cues increase PV levels through yet-to-be determined mechanisms. The absence of any changes in WFA intensity across time or after cocaine treatment given every other day for 3 days in the current study is similar to the absence of changes we previously observed after CPP testing in the drug-free state (Jorgensen et al., 2020).

### PNN Removal Reduces the Number of WFA-Surrounded PV Neurons but Does Not Alter the Number of PV Cells After Cocaine CPP Training or Cocaine-Primed Reinstatement

**Figure 3:**
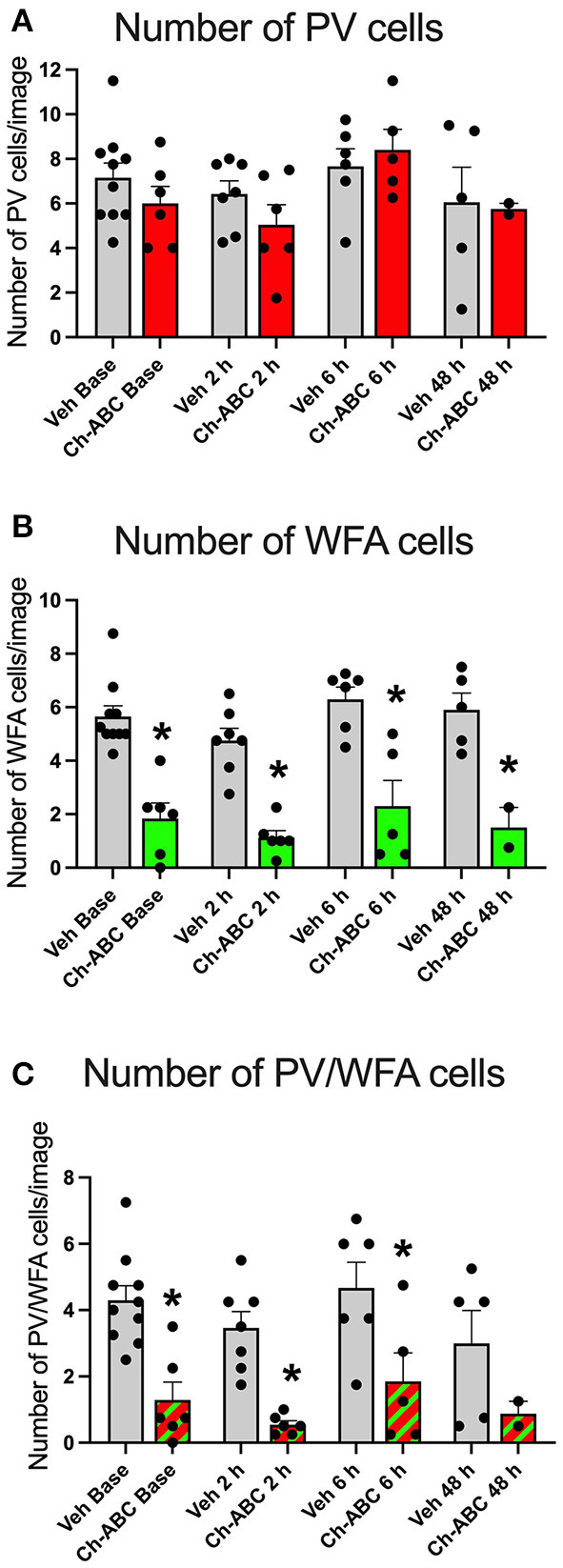
Cocaine-primed reinstatement does not alter the number of PV or WFA cells in the prelimbic PFC. **(A)** Number of PV cells in prelimbic PFC. **(B)** Number of WFA cells in prelimbic PFC; WFA cell number decreased in Ch-ABC treated animals at baseline, 2, 6, and 48 h compared to vehicle. **(C)** Number of PV/WFA double labeled cells in prelimbic PFC; PV/WFA cell number decreased at baseline, 2 h, and 6 h compared to vehicle. Bars show mean ± SEM, and individual data points depict the mean number of cells per rat across four images; N-sizes are as in Figure 2. *P < 0.05 compared to vehicle group at the same time point.

Ch-ABC alters PV firing by removing the controlled ionic microenvironment (Hartig et al., 1999) that allows PV cells to fire at high rates without succumbing to oxidative stress (Cabungcal et al., 2013). It is therefore possible that Ch-ABC treatment combined with cocaine, which itself increases oxidative stress (Kovacic, 2005) reduced the number PV cells. We therefore measured the number of PV and PNN cells in the prelimbic PFC. Figure 3A shows that there was no impact of treatment on the number of total PV cells within or between vehicle- and Ch-ABC-treated rats. A two-way ANOVA revealed no effect of treatment [F(1,39) = 0.579, *p* = 0.4514], time [F(2,39) = 2.499, *p* = 0.0738], or treatment x time interaction [F(3,39) = 0.579, *p* = 0.6322]. We also compared the number of WFA labeled cells, shown in Figure 3B. As expected, Ch-ABC treatment reduced the number of WFA labeled cells. A two-way ANOVA revealed a main effect of treatment [F(1,39) = 84.86, *p* < 0.0001] but not time [F(3,39) = 2.085, *p* = 0.118] or treatment x time interaction [F(3,39) = 0.119, p = 0.948). Ch-ABC treatment decreased the number of WFA labeled cells in all Ch-ABC-treated groups compared to vehicle controls (pre-reinstatement, *p* < 0.0001; 2 h, *p* < 0.0001; 6 h, *P* < 0.00081; 48 h, *p* = 0.0014). Similarly, Figure 3C shows that the number of WFA-surrounded PV cells was reduced after Ch-ABC treatment. A two-way ANOVA showed a main effect of treatment [F(1,39) = 31.55, *p* < 0.0001] but no effect of time [F(3,39) = 1.809, *p* = 0.1616], and no treatment x time interaction [F(3,39) = 0.125, *p* = 0.9446]. The number of PV cells surrounded by WFA were decreased in Ch-ABC-treated groups compared to vehicle controls at pre-reinstatement (*p* < 0.0017), 2 h (p = 0.005), and 6 h (*p* = 0.0015).

### PNN Removal Alters Excitability of FSIs After Cocaine-Primed Reinstatement

**Figure 4:**
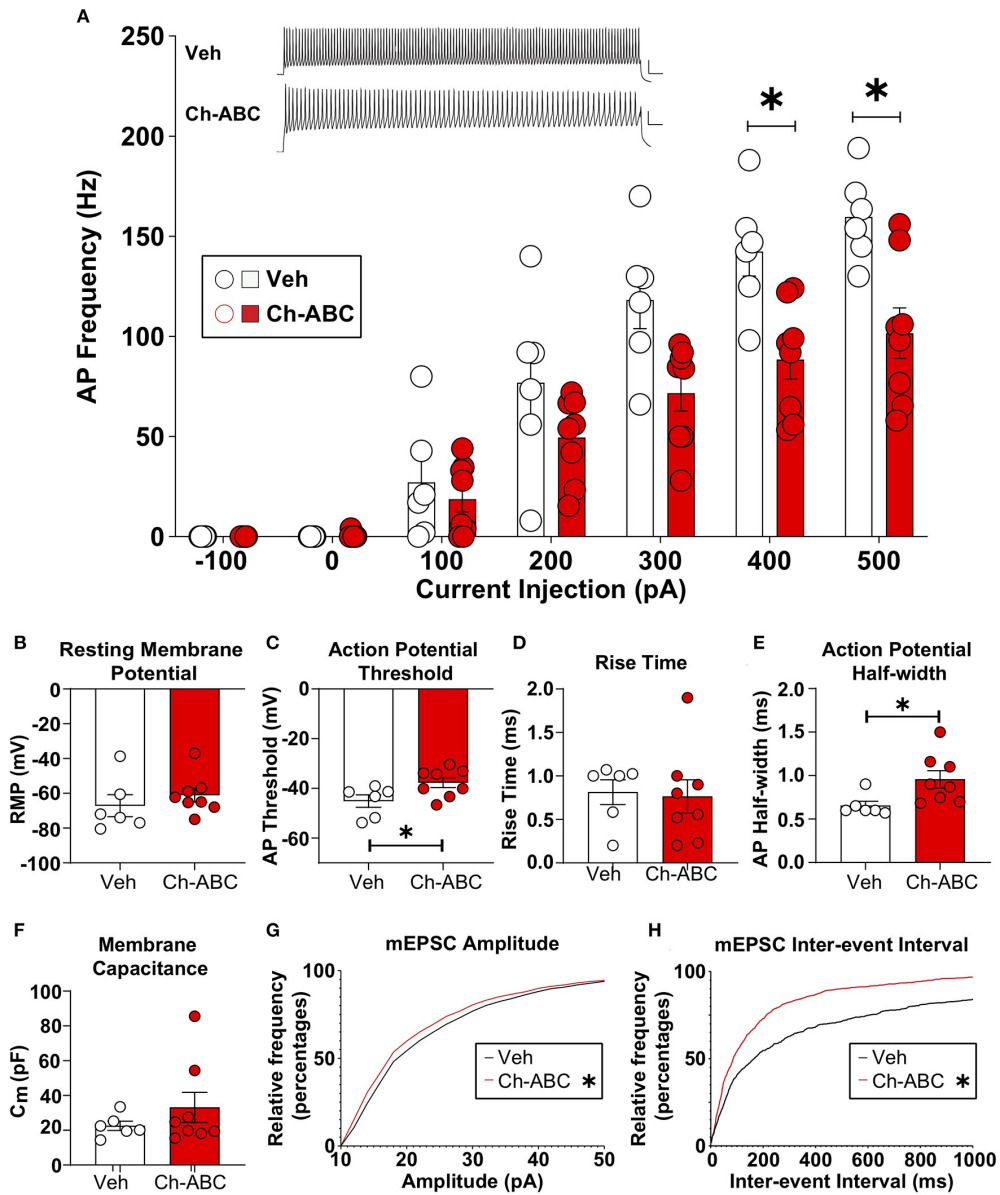
Ch-ABC treatment prior to cocaine-primed reinstatement decreases the firing rate of PNN surrounded FSIs within the prelimbic PFC and changes mEPSC frequency and amplitude onto FSIs. **(A)** Decrease in average frequency of action potentials across range of current injections; shown are representative traces of action potentials evoked by 500 pA current injection. Scale bars represents 20 ms, 20 mV; **(B)** Resting membrane potential; **(C)** Decrease in action potential threshold; **(D)** Rise time; **(E)** Increase in action potential half-width; **(F)** Membrane capacitance; **(A–F)**: Vehicle, n = 6 rats, Ch-ABC = 8 rats. **(G)** Decrease in mEPSC amplitude; **(H)** Increase in mEPSC frequency; Data are mean ± SEM (bars) with individual cells (circles). **(G,H)**: Vehicle n = 4 rats, Ch-ABC n = 5 rats; Mann-Whitney, *P < 0.05.

We examined how cocaine-induced reinstatement impacted the intrinsic properties of fast-spiking neurons, which contain PV (Kawaguchi and Kubota, 1993; Povysheva et al., 2008; Ferguson and Gao, 2018); hereafter referred to as FSIs) from vehicle and Ch-ABC-treated rats. We focused on the 2 h timepoint based on our previous findings that drug-free cocaine CPP memory retrieval produced the largest decrease in elicited action potentials in FSIs (Jorgensen et al., 2020). All electrophysiological results are summarized as the mean + SEM. Figure 4A shows that Ch-ABC infusion 3 days prior to cocaine-primed reinstatement attenuated the frequency of current-induced action potentials in FSIs compared with vehicle controls 2 h after reinstatement. A two-way ANOVA showed a main effect of treatment [F(1,12) = 7.53, *p* = 0.0178], current injection [F(2.024,24.28) = 132.2, *p* < 0.0001], and a treatment x current injection interaction [F(6,72) = 7.23, *p* < 0.0001]. Šídák's multiple comparisons test confirmed there was a significant difference at 400 pA (vehicle = 142.42 ± 12.26, *n* = 6; Ch-ABC = 88.46 ± 9.85, *n* = 8) and 500 pA (vehicle = 159.70 ± 9.1, *n* = 6; Ch-ABC = 101.58 ± 12.68). We did not find a change in resting membrane potential (Figure 4B) but did observe that 2 h after cocaine-primed reinstatement, Ch-ABC treatment altered several intrinsic membrane properties of FSIs. Ch-ABC treatment decreased the action potential threshold (Figure 4C; vehicle = −45.13 ± 2.51, Ch-ABC = −37.72 ± 2.00, t = 2.34, *p* = 0.0376) and increased the action potential half-width (Figure 4E; vehicle = 0.65 ± 0.05, Ch-ABC = 0.96 ± 0.10, t = 2.72, *p* = 0.0212). Several other intrinsic properties did not show significant changes: input resistance, action potential amplitude, afterhyperpolarization potential, first interspike interval, rheobase, rise time (Figure 4D), and capacitance (Figure 4F). Overall, these changes indicate that FSIs have a more depolarized membrane potential and thus may fire more easily at low current input, but that they cannot maintain high frequency firing, consistent with other studies (Balmer, 2016; Christensen et al., 2021).

### PNN Removal Increases Excitatory Synaptic Transmission Onto FSIs After Cocaine-Primed Reinstatement

For analysis of excitatory synaptic transmission, we analyzed the amplitude and inter-event interval of miniature excitatory postsynaptic currents (mEPSCs). In Figure 4, we show the cumulative probability frequency, which is more sensitive and representative of all miniature events. Ch-ABC treatment 3 d prior to cocaine-primed reinstatement attenuated the amplitude of mEPSCs onto FSIs compared with vehicle-treated controls 2 h after reinstatement (Figure 4G: *p* < 0.0001; Kolmogorov-Smirnov D = 0.0636). In addition, there was a decrease in inter-event interval, indicative of an increase in frequency of mEPSCs (Figure 4H: *p* < 0.0001; Kolmogorov-Smirnov D = 0.2001). This increased excitatory input may be compensatory for the changes we found in intrinsic properties (Campanac and Hoffman, 2013).

## Discussion

### PNN Removal Has Minimal Impact on Cocaine-Primed Reinstatement

We found that PNN removal did not significantly reduce the magnitude of cocaine-primed reinstatement compared with vehicle-treated rats, in agreement with our previous work in which the initial “memory reactivation” session was equivalent to the reinstatement session used here (Slaker et al., 2015). However, Ch-ABC-treated rats did not significantly increase the time spent in the cocaine-paired compartment after cocaine-primed reinstatement (*p* = 0.065). This slight attenuation in response could be due to a decreased ability to process cocaine-associated spatial or other cue information. Activation of groups of neurons, (neural ensembles), underlie memory retrieval (Agetsuma et al., 2018; Roy et al., 2022). We speculate that the presence of cocaine on the reinstatement day may produce interoceptive cues that activate additional neural ensembles to promote more extensive memory retrieval and support reinstatement behavior. Cocaine-induced increases in dopaminergic transmission may generate memory retrieval by activating discrete neural ensembles representing cocaine-associated memories through stimulation of PV neurons (Gorelova et al., 2002) that inhibit nearby pyramidal neurons.

### Cocaine Reinstatement Alters PV Intensity After PNN Removal

Within minutes to hours after memory retrieval, the memory is in a labile state (Nader et al., 2000; Sara, 2000). We did not observe changes in PV intensity in vehicle-treated rats after memory retrieval by cocaine-induced reinstatement. Our previous studies suggest that two key factors may regulate PV intensity in a bidirectional manner after cocaine-associated memory retrieval. First, we reported that five daily cocaine injections increased the intensity of PV in PV/WFA double-labeled cells by about 25% when examined 2 h after the last cocaine injection, with smaller increases retained 24 h later (Slaker et al., 2018). In a different study using cocaine-induced CPP, we found that placing rats in the CPP chamber for memory retrieval in the *drug-free state* had the opposite effect, with decreased PV levels across all time points, from 30 min to 24 h later (Jorgensen et al., 2020). These studies combined with our current results suggest that the *pharmacological effects* of cocaine elevate PV levels, while placement into the cocaine *context* decreases PV levels, but that when animals are exposed to both the context and cocaine as in the present study, opposing effects may result in no changes in PV intensity, with the exception of a small decrease 48 h later. Indeed, cocaine-induced dopamine levels would be expected to increase the excitability of PV neurons in the PFC (Gorelova et al., 2002). Notably, we measured PV intensity levels in the cell soma only, and PV is distributed throughout neurites. Cocaine exposure prenatally alters the distribution of PV within dendrites in the mPFC (Morrow et al., 2005) and the anterior cingulate cortex (Wang et al., 1996), so it is possible that a reduction in PV immunostaining within the soma could be due to redistribution within neurites.

In contrast to our findings in vehicle controls, when we removed PNNs with Ch-ABC, cocaine-primed reinstatement increased PV intensity of PV cells 2 h later compared with vehicle controls (Figure 2A). The intensity of PV in the remaining double-labeled PV/WFA cells was decreased compared with vehicle controls, as would be expected based on previous reports that PNN removal reduces PV intensity (Yamada et al., 2014), and PV intensity has been correlated with PNN intensity (Slaker et al., 2015). Surprisingly however, in Ch-ABC-treated rats, cocaine-primed reinstatement increased the intensity of remaining PV cells surrounded by PNNs 2 h later relative to pre-reinstatement PV intensity (Figure 2C). These findings suggest that, with reduced PNNs, cocaine exposure in cocaine CPP-trained rats still elevates PV intensity, but in a transient manner. The consequence of these transient changes in PV intensity after cocaine-primed reinstatement remain unknown. Low PV levels have been shown to increase synaptic facilitation (Caillard et al., 2000; Eggermann and Jonas, 2011), while high PV levels may have the opposite effect of lowering the release of GABA, as PV acts as a fast calcium buffer within nanodomains (Eggermann et al., 2011). Hence, PNNs may provide important control of the PV microenvironment and associated network to regulate neuronal function, which is impaired when much of the PNN is degraded with Ch-ABC.

### PNN Removal Prior to Cocaine Reinstatement Reduces PV Neuron Firing

Cocaine-induced reinstatement combined with PNN removal increased the firing threshold for action potential generation but also decreased the firing rate of FSIs (PV neurons) at higher current input. These findings are consistent with other studies showing that PNN removal by Ch-ABC or other manipulations that alter PNN components or that naturally lack PNN components (brevican) reduces the ability of PV neurons to fire at higher frequencies (Dityatev et al., 2007; Favuzzi et al., 2017; Hayani et al., 2018; Wingert and Sorg, 2021). In addition, cocaine self-administration produces a more depolarized resting membrane potential in mPFC PV neurons (Wright et al., 2021). These findings suggest that low-level inputs may more easily trigger PV neuron firing, but that these neurons are less responsive to higher current inputs and show a reduction in recurrent action potentials, which could impair short-term plasticity after cocaine-primed reinstatement and may underlie the decreased reconsolidation of cocaine CPP we previously reported (Slaker et al., 2015). Given this bidirectional impact of PNN removal on PV neuron firing at low vs. high current input, PNNs may allow PV neurons to optimize both low- and high-frequency inputs, but may do so differently within distinct circuits that mediate particular behaviors. We note that while the majority of PV neurons in the mPFC are surrounded by PNNs (Slaker et al., 2015), it is possible some PV neurons we assessed had very low PNN content and were thus not sampled in our population. Additionally, in Ch-ABC-treated rats, we cannot be certain that all PV neurons we sampled from in electrophysiology studies formerly contained PNNs, and this may have led to some of the greater variability we observed in this group for certain measures (e.g., action potential frequency, membrane capacitance).

PV neurons are key to synchronizing pyramidal neuron output into separate groups of activated neurons that code separate memories (Tonegawa et al., 2015; Ye et al., 2016). Cocaine-induced increases in dopamine may initially recruit neural ensembles by enhancing plasticity through D1 receptor-dependent mechanisms (Karunakaran et al., 2016), possibly *via* proteolysis of the PNN constituents brevican and aggrecan in the rat PFC (Mitlohner et al., 2020). This would support our findings that PNNs change in intensity following initial cocaine exposure (Slaker et al., 2018), leading to physiological changes and altering behavioral output. However, enzymatic PNN degradation decreases the excitability of PV neurons, which would disrupt their precise firing patterns and produce overlapping neuronal ensembles, leading to less specificity of which ensembles represent a particular stimulus or memory (Agetsuma et al., 2018; Christensen et al., 2021).

PV neurons are essential for generating gamma oscillations (Engel et al., 2001; Cardin et al., 2009; Sohal et al., 2009; Uhlhaas et al., 2010; Buzsaki and Wang, 2012; Stark et al., 2013). Therefore, any changes in PV levels or PV cell firing may impact circuits that depend on gamma activity. Gamma oscillations are required for attention (Kim et al., 2016; Bueno-Junior et al., 2017), adapting new strategies (Guise and Shapiro, 2017; Cho et al., 2020), and encoding reward outcomes (Donnelly et al., 2014) or expected outcomes (Del Arco et al., 2017). Based on previous studies examining the role of PV neurons in gamma oscillations, we speculate that, after PNN removal, decreased pre-reinstatement PV levels found in the remaining PV/PNN-containing cells would reduce gamma activity (Volman et al., 2011; Carceller et al., 2020), although transient increases in gamma activity occur in the adult visual cortex after monocular deprivation (Lensjo et al., 2017). PNN removal also increases the variability of firing that in turn could alter the tightly regulated timing needed to maintain functional gamma oscillations in response to environmental stimuli (Lensjo et al., 2017). Cocaine increases cortical surface gamma activity (Kiyatkin and Smirnov, 2010), and acute cocaine delivery increases optogenetically induced gamma oscillations through D1 receptors in the mPFC (Dilgen et al., 2013). Thus, impaired PV function after PNN removal may partially interfere with the ability of cocaine to enhance gamma oscillations. Disruption of these oscillations within or between brain regions may help explain our previous findings that memory retrieval in the absence of PNNs disrupted the reconsolidation of a cocaine CPP memory (Slaker et al., 2015). These findings are consistent with other studies showing the ability of PNN degradation to disrupt reconsolidation (Radiske et al., 2020) and to retain a stable grid cell network in a spatial environment (Christensen et al., 2021). Thus, PNNs support the fast-spiking ability of PV neurons needed for precise timing to generate gamma oscillations, and these oscillations within and between brain regions may underlie cocaine-enhanced gamma oscillations that lead to strongly encoded cocaine-associated memories.

### PNN Intensity Changes Are Minimal After Cocaine-Primed Reinstatement

Removal of PNNs with Ch-ABC generally reduced WFA intensity and the number of WFA cells as well as the number of PV/WFA double-labeled cells, as expected (Figures 2B,D). However, we found only a small decrease in WFA intensity in vehicle controls 48 h after cocaine-primed reinstatement and no differences in WFA intensity surrounding PV-labeled vs. non-PV-labeled neurons. These results were not unexpected, as we previously saw only relatively small increases in WFA intensity 2 h after five daily cocaine injections (Slaker et al., 2018) and no changes after drug-free re-exposure to the CPP apparatus after training for cocaine CPP (Jorgensen et al., 2020). We also did not observe differences in pre-reinstatement groups or at any time after cocaine-induced reinstatement in vehicle controls (Figure 2F). It is not yet apparent what other cell types (besides PV neurons) PNNs surround in the prelimbic PFC, as some PNNs surround pyramidal neurons, but this is highly variable throughout cortical regions (Alpar et al., 2006). It is also possible that PNNs surround only PV neurons in the prelimbic PFC, but that PV levels in some cells are below the detectable limit of our analysis.

Cocaine treatment and handling of rats during training, extinction, and the reinstatement session may have disrupted normal circadian rhythmicity in our rats (Jansen et al., 2012). We recently reported diurnal rhythmicity in the intensity of PNNs and PV (Harkness et al., 2021). However, in the absence of additional studies, it is not possible to know if the impact of cocaine reinstatement on PV/PNN intensity reported here was altered by the natural diurnal rhythms of PV and PNNs, but future studies should examine how cocaine administration at different times of day alters these rhythms.

## Conclusions

Our previous work showed that cocaine-induced reconsolidation of a cocaine CPP memory was dependent on the presence of PNNs in the prelimbic PFC (Slaker et al., 2015). Here we delineated the dynamic cellular and electrophysiological changes in prelimbic PFC PV neurons after memory retrieval and during the reconsolidation period following cocaine-primed CPP reinstatement in the presence and absence of PNNs. We found that PNN removal by Ch-ABC 3 days prior to cocaine-primed reinstatement somewhat dampened this response, although it was not different from vehicle-treated controls on the reinstatement day. The Slaker et al. (2015) study demonstrating no impact on initial cocaine-primed memory retrieval (equivalent to the reinstatement session used here) and the current work showing no major differences in cocaine-primed reinstatement between control and Ch-ABC-treated rats suggest that Ch-ABC does not alter initial memory retrieval but does interfere with the reconsolidation of cocaine memory when retrieval occurs in the absence of PNNs. Cocaine-primed reinstatement in vehicle-treated rats had little to no effect on the number or intensity of PV cells, with exception of a small decrease in PV intensity 48 h later. However, PNN removal with Ch-ABC, while decreasing the intensity of the remaining PV neurons surrounded by PNNs prior to reinstatement, rendered these neurons vulnerable to cocaine-induced increases 2 h after cocaine-primed reinstatement. Interestingly, this effect of cocaine-primed reinstatement on PV intensity was nearly identical to that of PV neurons that were naturally devoid of PNNs in vehicle controls. Slice electrophysiology studies showed that at 2 h post-reinstatement, Ch-ABC reduced the excitability of PV neurons. We found no changes in the intensity of WFA but the expected severe reduction in number of WFA-surrounded neurons. Overall, these findings show that PNNs may be protective of cocaine-induced changes in PV intensity, and these changes in intensity after Ch-ABC treatment may in part prevent subsequent memory reconsolidation. The fast-spiking activity of PV neurons supported by the presence of PNNs likely underlies the ability of cocaine to enhance gamma oscillations that may promote strong cocaine-associated memories and may also underlie the inability to maintain these memories when PNNs are degraded.

## Data Availability Statement

The raw data supporting the conclusions of this article will be made available by the authors, without undue reservation.

## Ethics Statement

The animal study was reviewed and approved by the Institutional Animal Care and Use Committees at Washington State University, Legacy Research Institute, and the University of Wyoming in accordance with the National Institute of Health Guide for the Care and Use of Laboratory Animals.

## Author Contributions

AG, EJ, JR, JH, and JA collected data, generated images, and performed analyses on the data. TB and BS conceived and designed the study. AG, EJ, TB, and BS wrote the manuscript. All authors contributed to manuscript revision and approved the submitted version.

## Funding

This work was supported in part by National Institutes of Health (NIH) DA047121 (BS) and DA040965 (BS and TB). Other support was from the Good Samaritan Foundation of Legacy Health (BS).

## Conflict of Interest

JH is the CEO of Rewire Neuro. The remaining authors declare that the research was conducted in the absence of any commercial or financial relationships that could be construed as a potential conflict of interest.

## Publisher's Note

All claims expressed in this article are solely those of the authors and do not necessarily represent those of their affiliated organizations, or those of the publisher, the editors and the reviewers. Any product that may be evaluated in this article, or claim that may be made by its manufacturer, is not guaranteed or endorsed by the publisher.
